# Reciprocal Modulation of I_K1_–I_Na_ Extends Excitability in Cardiac Ventricular Cells

**DOI:** 10.3389/fphys.2016.00542

**Published:** 2016-11-15

**Authors:** Anthony Varghese

**Affiliations:** Department of Computer Science, University of Wisconsin-River FallsRiver Falls, WI, USA

**Keywords:** reciprocal modulation, cardiac cells, mathematical model

## Abstract

The inwardly rectifying potassium current (I_K1_) and the fast inward sodium current (I_Na_) are reciprocally modulated in mammalian ventricular myocytes. An increase in the expression of channels responsible for one of these two currents results in a corresponding increase in expression of the other. These currents are critical in the propagation of action potentials (AP) during the normal functioning of the heart. This study identifies a physiological role for I_K1_–I_Na_ reciprocal modulation in ventricular fiber activation thresholds and conduction. Simulations of action potentials in single cells and propagating APs in cardiac fibers were carried out using an existing model of electrical activity in cardiac ventricular myocytes. The conductances, G_K1_, of the inwardly rectifying potassium current, and G_Na_, of the fast inward sodium current were modified independently and in tandem to simulate reciprocal modulation. In single cells, independent modulation of G_K1_ alone resulted in changes in activation thresholds that were qualitatively similar to those for reciprocal G_K1_–G_Na_ modulation and unlike those due to independent modulation of G_Na_ alone, indicating that G_K1_ determines the cellular activation threshold. On the other hand, the variations in conduction velocity in cardiac cell fibers were similar for independent G_Na_ modulation and for tandem changes in G_K1_–G_Na_, suggesting that G_Na_ is primarily responsible for setting tissue AP conduction velocity. Conduction velocity dependence on G_K1_–G_Na_ is significantly affected by the intercellular gap junction conductance. While the effects on the passive fiber space constant due to changes in both G_K1_ and the intercellular gap junction conductance, G_gj_, were in line with linear cable theory predictions, both conductances had surprisingly large effects on fiber activation thresholds. Independent modulation of G_K1_ rendered cardiac fibers inexcitable at higher levels of G_K1_ whereas tandem G_K1_–G_Na_ changes allowed fibers to remain excitable at high G_K1_ values. Reciprocal modulation of the inwardly rectifying potassium current and the fast inward sodium current may have a functional role in allowing cardiac tissue to remain excitable when I_K1_ is upregulated.

## Introduction

The inwardly rectifying potassium current (I_K1_) sets the resting membrane potential of atrial and ventricular cardiac myocytes while the fast inward sodium current (I_Na_) determines the rate of depolarization and speed of propagating action potentials (APs) in tissue. These two currents have opposing effects on cardiac excitability: increasing the conductance, G_K1_, of I_K1_ is thought to decrease excitability by raising the threshold needed to initiate an AP while increasing the conductance, G_Na_, of I_Na_ is believed to increase excitability mainly by facilitating depolarization. The conductance values of ionic currents reflect the number of functional channels in the cell membrane and, therefore, alterations in conductances represent long-term changes that occur in cardiac cells and tissue. The term “reciprocal modulation” refers to the recent finding (Milstein et al., 2012) in mammalian heart cells that upregulation of channel density of one current (I_K1_ or I_Na_) results in a parallel upregulation of the other current. One question that arises from the data of Milstein et al. (2012) is why heart cells would have such a regulatory mechanism: does reciprocal modulation have a role in cardiac excitability*?* Milstein et al. discuss the importance of reciprocal modulation in potentially fatal cardiac rhythm disturbances but such a function would be unfavorable to an organism. A cardioprotective role for reciprocal modulation would provide an evolutionary advantage and, therefore, a teleological reason for the existence of this important biological phenomenon. While a number of modeling studies have examined the effect of changes in individual conductances in heart cells, the effect of tandem changes in I_K1_ and I_Na_ have not yet been fully explored. The central hypothesis of this study is that the reciprocal modulation of I_K1_ and I_Na_ discovered by Milstein et al. (2012) is a mechanism that cardiac cells use to regulate excitability.

Cardiac ion channels are organized and targeted to specific cellular locations by numerous cardiac-specific membrane and structural proteins (Balse et al., 2012). Kucera et al. (2002) showed that Na_v_ 1.5 (also referred to as “rH1” in rat cells) channels, responsible for much of the fast inward sodium current in mammalian ventricular myocytes, co-localize at cardiac cell intercalated disks (ID) with Connexin-43 (Cx43), the molecule primarily responsible for the intercellular gap junction current, I_gj_, in ventricular tissue. Cx43 is linked biochemically to ZO-1, the zona occludens-1 protein found in intercalated disks, and was shown by Barker et al. (2002) to be regulated by ZO-1. Ankyrin-G, which is also expressed in cardiac cell intercalated disks (Mohler et al., 2004), plays a role in anchoring Nav1.5 (Lowe et al., 2008) at IDs. Milstein et al. (2012) showed that Kir2.1, which is responsible for I_K1_ in ventricular cells, and Nav1.5 channels interact functionally within a macromolecular complex involving the SAP97 scaffolding protein localized to cardiac gap junctions; the Kir2.1–Nav1.5 interaction was referred to as “*reciprocal modulation*” by Milstein et al. (2012) because experimental interventions that increase the channel density of one current were found to increase the other. Their data (Milstein et al., 2012, Figures 1, 2) showed no significant changes in channel gating or voltage dependence and while they did see hyperpolarizing shifts in both sodium channel activation and inactivation in one case (Milstein et al. **Figure 4**), it is likely that the larger shift in the voltage dependence of inactivation will result in an overall reduced excitability due to changes in Nav1.5 gating. The primary effect of reciprocal modulation appears to be that of parallel changes in channel densities. These results suggest some amount of cooperativity between I_K1_, I_Na_, and I_gj_. Recently, Varghese et al. (2015) showed, using a combination of simulations and experiments, that the mode of action of therapeutic doses of class I antiarrhythmic agents can be understood by analyzing the three currents involved in initiating cardiac APs: I_K1_, I_Na_, and I_gj_. This paper focuses on the changes in excitability due to independent and reciprocal modulation of the I_K1_–I_Na_ conductances.

**Figure 1:**
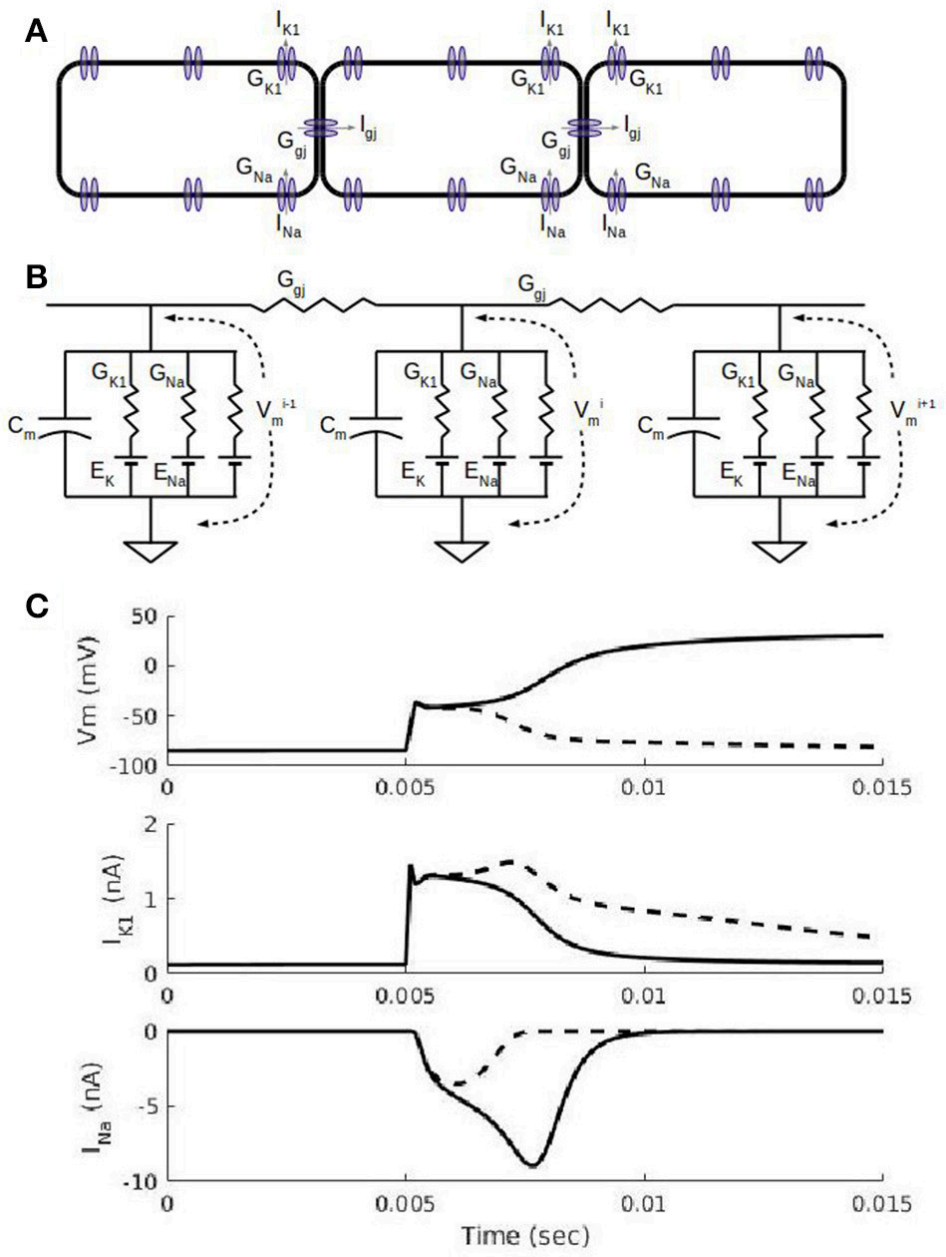
**Action potential propagation in a cardiac fiber. (A)** Schematic view of three cells in a fiber. In addition to a number of sodium, potassium, and calcium currents in the cell membrane, each cell has non-selective gap junction channels that allow currents to flow down the fiber. The gap junction current, I_gj_, allows a depolarized cell to drive electrical activity in a neighboring cell. **(B)**. Equivalent circuit representation of the three cells in **(A)**. Each cell has a capacitance associated with the cell membrane and ionic currents represented by a conductance and a battery for the Nernst potential for the corresponding ion. The inward rectifier potassium current, I_K1_, current has a non-linear conductance (see Methods) with a maximal conductance, G_K1_, and its reversal potential is the potassium Nernst potential, E_K_. The fast sodium current, I_Na_, has a time-dependent conductance with the maximal conductance, G_Na_, and its reversal potential is the sodium Nernst potential, E_Na_. **(C)** Simulations showing cell membrane potential, V_m_, and ion channel currents, I_K1_ and I_Na_, for a subthreshold stimulus (dashed curves) and suprathreshold stimulus (solid curves). Results shown are for the 5th cell in a fiber of 100 cells with 0.2 ms-duration current stimuli administered at the 5 ms mark to the first 4 cells. A subthreshold stimulus brings V_m_ to about −60 mV after which V_m_ declines (dashed curve) back to its resting membrane potential of about −90 mV; the I_K1_ waveform due to a subthreshold stimulus is larger than the suprathreshold case (solid curve) because the subthreshold V_m_s are more negative and thus less inward rectified. I_Na_ is partially activated (dashed curve) for a subthreshold stimulus and fully activated (solid curve) for the suprathreshold stimulus; the fully activated I_Na_ is what triggers the full depolarization of V_m_ to about +20 Mv.

**Figure 2:**
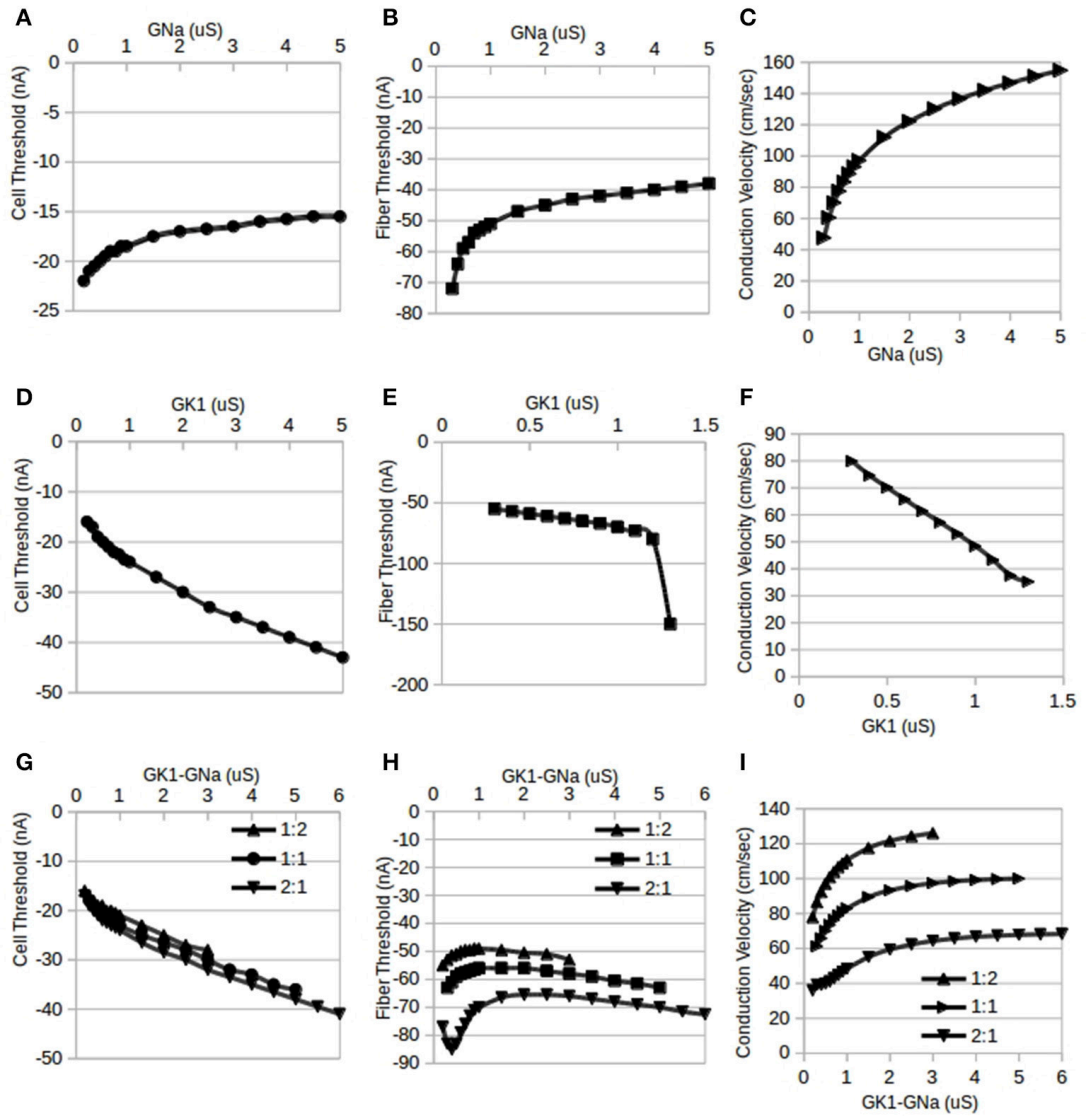
**Independent and reciprocal modulation of I_**K1**_ and I_**Na**_ in single cells and fibers**. By standard convention, inward (depolarizing) stimulus currents are negative. **(A)**. Changes in single cell threshold due to independent modulation of G_Na_. These results indicate that as G_Na_ is increased, the magnitude of the threshold is lowered. G_K1_ was held at its default value of 0.5 μS. **(B)** Fiber threshold was also lowered as G_Na_ was increased. G_K1_ was held at 0.5 μS and G_gj_ was held at 10 μS. **(C)** Fiber conduction velocity increased as G_Na_ was increased (same conditions as **B**). **(D)** Changes in single cell threshold due to independent modulation of G_K1_: as G_K1_ is increased, the magnitude of the threshold is raised. G_Na_ was held at 0.5 μS. **(E)** Fiber threshold was raised as G_K1_ was increased. For values of G_K1_ larger than 1.3 μS, the fiber was inexcitable. G_Na_ was held at its default value of 0.5 μS and G_gj_ was held at 10 μS. **(F)** Fiber conduction velocity decreased as G_K1_ was increased (same conditions as **D**). **(G)** Changes in single cell threshold due to reciprocal modulation of G_K1_ and G_Na_. Cell threshold increased monotonically with the tandem changes in G_K1_:G_Na_. Three different ratios for G_K1_:G_Na_ were used: 1:2, 1:1, and 2:1; the X-axis shows the G_K1_ values, the corresponding G_Na_ value depends on the ratio used. **(H)** Fiber threshold changes due to reciprocal modulation of G_K1_ and G_Na_. Three different ratios for G_K1_:G_Na_ were used (1:2, 1:1, and 2:1) while G_gj_ was held at 10 μS. The X-axis shows the G_K1_ values, the corresponding G_Na_ value depends on the ratio used. **(I)** Fiber conduction velocity increased with reciprocal modulation of G_K1_ and G_Na_ (same conditions as **D**). The X-axis shows the G_K1_ values, the corresponding G_Na_ value depends on the ratio used.

The dynamics of I_K1_ & I_Na_ are key to understanding cardiac action potential propagation characteristics. Figure 1A shows a schematic view of three neighboring cells in a fiber with I_K1_, I_Na_, and I_gj_ channels shown in the cell membrane. Although there are numerous other channels in cardiac cells which are included in the simulations in this paper, they are set to their default parameter values and are not of particular interest in this paper. An equivalent circuit diagram for the three cells is shown in Figure 1B with linear resistors modeling intercellular gap junction connexin channels. Figure 1C shows two short simulations of changes in the cell membrane voltage (V_m_), I_K1_, and I_Na_ in one cell in a fiber due to 0.2 ms duration stimuli applied at the 5 ms point: one simulation shows the result of a suprathreshold stimulus (solid curves) and the other is for a subthreshold (dashed curves) one. At cardiac resting potentials (around −85 mV, first 5 ms of top trace in Figure 1C), the cell membrane voltage, V_m_, is close to the reversal potential for potassium, E_K_, and even though Kir2 channels (responsible for I_K1_) are open at these potentials, the lack of driving force for potassium ions results in I_K1_ being close to 0 as can be seen in the first 5 ms of the middle traces in Figure 1C. An inward current due to an external current stimulus (as was used in simulations in Figure 1C) or current from neighboring cells through gap junction Cx43 channels will depolarize the cell, increasing the driving force for potassium ions and causing an outward flow of potassium which will tend to repolarize the cell. The dashed curves in Figure 1C show the result of a subthreshold stimulus: V_m_ returns to the resting potential, I_K1_ is large and I_Na_ is only partially activated. The solid curves in Figure 1C show the result of a suprathreshold stimulus: when the stimulus is sufficient to depolarize the cell to about −60 mV, the inward (negative) I_Na_ through Nav1.5 channels can be fully activated (solid curve, bottom trace of Figure 1C), causing further depolarization. Cell depolarization above −40 mV inactivates I_Na_, making it decrease in magnitude. Meanwhile, the inward rectification property of Kir2 channels will result in I_K1_ decreasing with additional depolarization above −50 mV. Ventricular cells typically reach a maximum potential of about 20 mV at which point, both I_K1_ and I_Na_ are minimal due, respectively, to inward rectification and inactivation. Depolarization of one cell in a fiber results in current (I_gj_ in Figure 1A) flowing downstream through Connexin43 (Cx43) channels to a neighboring cell, causing the same sequence of events as described above in the neighbor cell and in this way causing the AP front to propagate through the fiber. The relation between the simulations used in this paper and predictions from linear and non-linear cable theory is summarized in the Appendix.

Recent studies have shown evidence for spatial localization of Nav1.5, Kir2.1, and Cx43 channels at the intercalated disks (IDs) in cardiac myocytes using both immunofluorescence and computational modeling. Kucera et al. (2002) showed that Nav1.5 channels co-localize with Cx43 in IDs and their simulations, which included a model of the intercellular cleft space, suggested that such co-localization may have a protective role under conditions of greatly reduced coupling. Simulations of ephaptic coupling by Mori et al. (2008) have also generalized the cable model and shown how this mechanism may play a role under conditions of reduced gap junction coupling. The experimental and modeling results of Veeraraghavan et al. (2016) also suggest a role for Nav1.5 and Kir2.1 in intercellular communication through an ephaptic coupling mechanism in addition to gap junctions, myocyte geometry, and tissue architecture; by comparing a “*uniform*” model where ion channels are uniformly distributed with a “*polarized*” model where ion channels are spatially localized, it was shown that the polarized model was able to better explain anisotropic conduction patterns. Wei et al. (2016) also used an ephaptic coupling model to study anisotropic phenomena under conditions of reduced gap junction coupling. While ephaptic coupling could play a role in reciprocal modulation under conditions of reduced gap junction coupling, in order to simplify computations, it was not used here as the majority of the simulations involved high gap junction conductance values. The most practical approach for investigating reciprocal modulation appears to be monodomain simulations of cardiac fibers to construct hypotheses that may be tested *in vitro* in the future. This study examines the effects of independent modulation of G_K1_ & G_Na_ and reciprocal G_K1_–G_Na_ modulation on single-cell thresholds, fiber current thresholds, fiber conduction velocities and fiber space constants along with effects of changes in fiber G_gj_.

## Methods

A number of cardiac cell models are currently available and could be used to study reciprocal modulation. The guinea-pig ventricular myocyte model of Noble et al. (1998) was used here for simulations of single cells and one-dimensional cardiac fiber for a number of reasons: first, it is a well-established model of cells at physiological temperatures (37°C) that has been found to reproduce experimental data from isolated cells and cardiac fibers (Varghese et al., 2015). Secondly, it has the convenient feature that the I_Na_ and I_K1_ conductances were already the same: 0.5 μS and thus required no scaling or baseline adjustments for the study of reciprocal modulation. Other models were found to have very different values for G_K1_ and G_Na_ and while they could be scaled by single channel conductance (which are roughly the same for Kir2.1 homotetramers and Nav1.5), most other models have baseline parameters that yield very different channel densities for Kir2.1 and Nav1.5. Third, the guinea-pig ventricular myocyte has an action potential morphology that is closer to those in larger mammals and may make it easier to apply the results of guinea-pig studies to cardiac excitability in humans.

Each cardiac cell involves a set of 22 simultaneous non-linear differential equations listed in the aforementioned references. For the sake of brevity, the full set of equations are not shown but a few key currents are discussed here. The inward rectifier current, I_K1_, was modeled using the formulation:

$$\begin{array}{l} I_{K1}=G_{K1}\frac{{\left[K\right]}_{o}}{{\left[K\right]}_{o}+k_{mk1}}\frac{\left(V-E_{K}\right)}{1\text{+}e^{\frac{V_{m}-E_{K}-10}{RT/2F}}} \end{array}$$

where [*K*]_*o*_ is the external potassium concentration and *E*_*K*_ is the Nernst reversal potential for potassium, the other parameters are listed in the aforementioned references. The fast inward current, I_Na_: $I_{Na}=G_{Na}m^{3}h\left(V-E_{Na}\right)$ involves two differential equations for the kinetics of activation (*m*) and inactivation (*h*). The default value for both *G*_*K*1_ and *G*_*Na*_ was 0.5 μS (micro Siemens); the default value of the intercellular gap junction coupling conductance, G_gj_, was set to 10 μS in order to obtain a baseline conduction velocity of 70 cm/s. Fibers had uniform parameter values: there were no spatial variation in parameters. The standard notation of outward currents having positive sign is used throughout this paper: a negative current value indicates that the current is an inward one.

The gap junction coupling currents flowing into the *i-th* cell (see Figures 1A,B) are:

$$\begin{array}{lll} I_{gj}^{\left[i-1\right]} & = & -G_{gj}\left(V_{m}^{\left[i-1\right]}-V_{m}^{\left[i\right]}\right)\\ I_{gj}^{\left[i\right]} & = & G_{gj}\left(V_{m}^{\left[i\right]}-V_{m}^{\left[i+1\right]}\right) \end{array}$$

These gap junction currents are combined with the cell capacitance current and the total ion channel current in cell *i* to arrive at the differential equation involving the cell membrane potential for each cell:

$$\begin{array}{l} C_{m}\frac{dV_{m}^{\left[i\right]}}{dt}=I_{gj}^{\left[i-1\right]}-I_{gj}^{\left[i\right]}-I_{ion}^{\left[i\right]} \end{array}$$

Boundary conditions at both ends were Neumann or “no-flux” or “sealed end” boundary conditions. Thus, a chain of 100 cells in a fiber is, in essence, a system of 2200 differential equations that are solved simultaneously using standard numerical ordinary differential equation methods. The Matlab (Mathworks, Natick, MA, USA) variable-order variable-step size stiff differential equation solver ode15s with a default absolute tolerance of 1e-6 was used to compute numerical solutions.

Stimulus current thresholds were computed separately for each specified set of parameters. Single cells were stimulated at 1 Hz intervals using 0.2 ms duration square pulses whose magnitudes were increased in 0.5 nA increments until an action potential was successfully elicited by two consecutive pulses. The threshold for a fiber was computed by stimulating the first four cells in a 100 cell chain at 1 Hz intervals using 0.2 ms duration square pulses of increasing magnitude until two consecutive stable propagating APs were seen. The short pulse duration was selected so that there would be minimal effect on sodium activation and inactivation (the fast sodium current peaks in <1 ms and inactivates about as quickly). In addition, this stimulus duration is practical in patch-clamp and tissue experiments (Varghese et al., 2015). Conduction velocity simulations involved triggering a propagating AP with a current stimulus (in four cells at one end of the fiber) that was, by convention, 1.5 times the threshold to avoid artifacts due to propagation delays. By symmetry (Rall, 1977; Crawford et al., 1991), stimulating one end with sealed end boundary conditions is equivalent to stimulating in the middle of a fiber that is twice as long. Conduction velocities were measured by dividing the difference in the activation times at the 75th and 25th cells in the fiber by the distance in order to avoid end effects such as slower initiation of waves at the stimulus end and wave speed-up at the distal end due to the sealed-end boundary condition. The length, L_c_, of each cell was 74 μm. Fiber space constants were computed using long-duration subthreshold stimulus pulses administered at one end of the chain. These 100 ms pulses were long enough in time duration for the fiber to reach steady state and large enough in pulse magnitude (within 10% below the threshold) to allow accurate fitting to an exponential function. As in Noujaim et al. (2007) and Seidel et al. (2010), the voltages of the cells at the end of the pulse were fitted to a single exponential decay function. The Matlab lsqcurvefit least squares method was used to determine the rate of spatial decay, i.e., the space constant, as a multiple of cell lengths.

## Results

**Figure 3:**
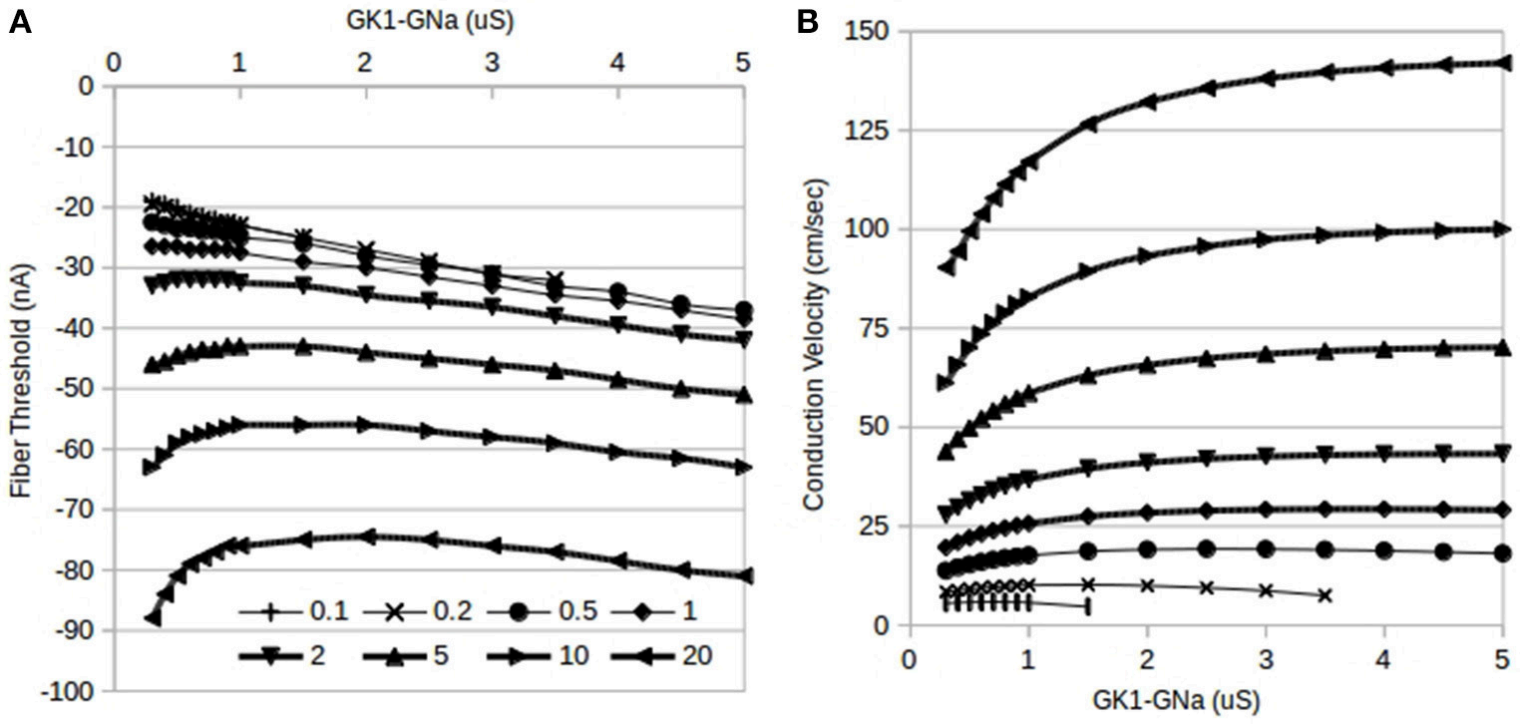
**Effects of reciprocal modulation of I_**K1**_ and I_**Na**_ and changes in gap junction conductance, G_**gj**_, on fiber thresholds**. **(A)** Changes in fiber threshold due to reciprocal modulation of G_K1_–G_Na_ (1:1 ratio). At any particular value of G_K1_–G_Na_, increasing G_gj_ raises the threshold for initiating a propagating action potential. The curves for G_gj_ = 0.1 and 0.2 μS (top two curves) overlap considerably. Magnitudes of current thresholds increase monotonically with increasing G_K1_–G_Na_ for G_gj_ = 0.1–1 μS while higher values of G_gj_ result in biphasic dependence. **(B)** Changes in fiber conduction velocity due to reciprocal modulation of G_K1_–G_Na_. At any particular value of G_K1_–G_Na_, increasing G_gj_ increases the conduction velocity of propagating action potentials. Markers are the same as panel A: top curve is for G_gj_ = 20 μS.

**Figure 4:**
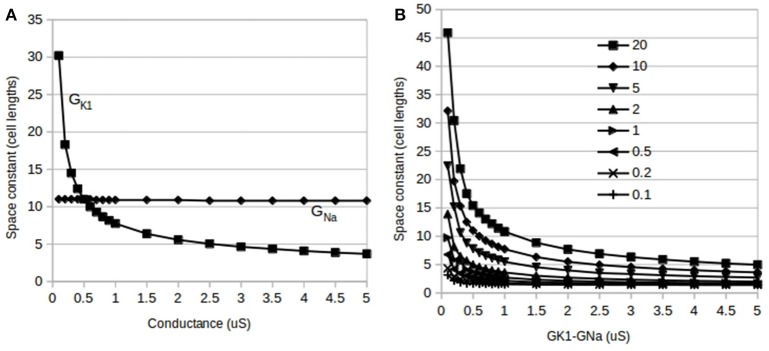
**Effects of independent and reciprocal modulation of I_**K1**_ and I_**Na**_ and changes in gap junction conductance, G_gj_, on fiber space constant**. **(A)** Changes in fiber space constant due to independent modulation of G_K1_ and G_Na_: the space constant is insensitive to changes in G_Na_ alone (G_K1_ held at 0.5 μS–flat line) but is very sensitive to alterations in G_K1_ (G_Na_ held at 0.5 μS–curve). **(B)** Changes in fiber space constant due to reciprocal modulation of G_K1_–G_Na_ (1:1 ratio). Each curve is for a different value of G_gj_ from 0.1 to 20 μS.

The results of this study show how excitability in single cardiac cells and fibers changes as ionic current conductances were modified. Parameter sweeps were conducted in two main ways: one was independent modulation where only a single conductance (either G_K1_ or G_Na_) was changed and the other was reciprocal modulation where both, G_K1_ & G_Na_, were modified in tandem. Figure 2 summarizes the results of modifying G_K1_ & G_Na_ independently (Figures 2A–F) and reciprocally (Figures 2G–I) in single cells (Figures 2A,D,G) and fibers (Figures 2B,C,E,F,H,I). Figure 3 shows the effects of reciprocal modulation for a parameter sweep of G_gj_ values and Figure 4 shows the results of independent (Figure 4A) and reciprocal (Figure 4B) modulation and G_gj_ changes on fiber space constant.

### Single cell threshold

Cells were stimulated using a 0.2 ms duration square pulse. At the default value of G_K1_ = G_Na_ = 0.5 μS, the computed single cell threshold was approximately −19 nA, matching observations in patch-clamp experiments with isolated guinea-pig cardiac ventricular myocytes using the same square pulse duration (Varghese et al., 2015). As shown in Figure 2A, holding the conductance, G_K1_, of the I_K1_ current at the default value of 0.5 μS and increasing G_Na_ from 0.2 to 5 μS, we see, as expected, that higher values of G_Na_ lowers the magnitude of the inward current needed to trigger an action potential (AP). Figure 2D shows that with G_Na_ held at its default value of 0.5 μS and G_K1_ varied from 0.3 to 5 μS, the magnitude of the threshold is raised monotonically by increasing G_K1_. Values of G_K1_ lower than 0.3 μS resulted in action potential durations (APD) >1 s and were, therefore, not considered while values higher than 5 μS resulted in extremely short APDs, on the order of ms and were not considered realistic. Baseline observations showing that independent increments in G_Na_ lowers the threshold while increments in G_K1_ raises it agree with expectations: since I_Na_ is an excitatory current and I_K1_ inhibits APs, increasing G_Na_ would be expected to make it easier to APs while increasing G_K1_ has the opposite effect. This poses the question of which trend will be seen with reciprocal modulation when G_K1_ and G_Na_ are both incremented in tandem: will one current dominate or will they neutralize each other?

Figure 2G shows the effect of reciprocal modulation on single cell thresholds: even though the actual magnitude of I_K1_ is lower than that of I_Na_, it is G_K1_ that primarily determines the threshold for cellular activation. In the competition between the opposing conductances, G_K1_ and G_Na_, it is G_K1_ that “wins.” While Milstein et al. (2012) did have quantitative data on reciprocal modulation, they did not predict a particular ratio for the changes in G_K1_ and G_Na_. Simulations using G_K1_:G_Na_ stoichiometries other than 1:1 reciprocal modulation, such as 2:1 and 1:2 (Figure 2G; the x-axis shows the G_K1_ values, the corresponding G_Na_ value depends on the ratio used) reveal that the overall trends were qualitatively very similar to that seen for 1:1 reciprocal modulation: increasing G_K1_ and G_Na_ in tandem raises the threshold for activation.

### Fiber threshold

Chains of 100 coupled (G_gj_ = 10 μS) cells were stimulated using 0.2 ms square pulses applied to four cells at one end of the fiber. Figure 1C shows the cell V_m_, I_K1_, and I_Na_ for the 5th cell in the fiber for two such simulations: one for a subthreshold stimulus and one for a suprathreshold stimulus. While similar results would be expected for the dependence of the thresholds in single cells and fibers, simulation results show some important differences. Figure 2B shows the trend in stimulus threshold in a fiber as the G_K1_ of each cell was held at the default value of 0.5 μS and G_Na_ was increased from 0.2 to 5 μS. As in the case of single cells (Figure 2A), we see that an increase in G_Na_ lowers the magnitude of the threshold required to initiate a propagating AP; as expected, larger current stimuli are needed to initiate APs in fibers vs. single cells.

Figure 2E shows that with G_Na_ held at its default value of 0.5 μS while G_K1_ is increased from 0.3 μS, the magnitude of the threshold is raised monotonically by increasing G_K1_. As with single cells, the fiber does not allow the use of values of G_K1_ below 0.3 μS since the APD of each cell exceeds 1 s. A crucial difference between the single cell case and the fiber is that at values of G_K1_ above 1.2 μS, the fiber threshold increases dramatically to the point where the fiber is rendered inexcitable for G_K1_ > 1.3 μS.

Unlike the results of independent modulation of G_K1_, reciprocal modulation of G_K1_ and G_Na_ results (Figure 2H; the x-axis shows the G_K1_ values, the corresponding G_Na_ value depends on the ratio used) in the fiber remaining excitable for a significantly larger range of parameter values. Furthermore, the shapes of the curves in Figure 2H are qualitatively different from those for single cells (Figure 2G). Rather than the monotonically increasing or decreasing curves seen in Figures 2A,B,D,E,G, the fiber threshold is more complicated: a biphasic dependence on G_K1_/G_Na_ is seen for 1:1 and 1:2 stoichiometries while the results for 2:1 reciprocal modulation show a triphasic dependence. Thus, we cannot say that G_K1_ alone determines the fiber threshold. The interaction of the three currents, I_K1_, I_Na_, and I_gj_, with reciprocal modulation reveal behavior that is more complicated than predictions either from single cells or from independent modulation. These results in Figures 2E,H suggest that cells with larger G_K1_ become inexcitable unless accompanied by some increase in G_Na_. One interpretation may be that reciprocal modulation of the two opposing currents allows the threshold to stay within a tighter set of limits while also allowing ventricular tissue to remain excitable.

### Conduction velocity

The conduction velocities of propagating action potentials in fibers were studied under conditions of independent modulation (Figures 2C,F) and reciprocal modulation (Figure 2I; the x-axis shows the G_K1_ values, the corresponding G_Na_ value depends on the ratio used). It would be expected that G_K1_ would decrease excitability and, thus, conduction velocities as well while G_Na_ would have the opposite effect and that was precisely what was observed in simulation results. Figure 2C shows that, as predicted by non-linear cable theory (see Appendix), conduction velocity increases with independent modulation of G_Na_ from 0.3 to 5 μS while G_K1_ was held at its default value of 0.5 μS. Figure 2F shows that conduction velocity decreases as G_K1_ is increased from 0.2 to 1.3 μS while G_Na_ was held at 0.5 μS. As explained above, independent modulation of G_K1_ results in inexcitable tissue for values of G_K1_ above 1.3 μS. One remaining question is which of the two opposing currents will determine conduction velocity changes for the case of reciprocal modulation. Figure 2I shows that for all three stoichiometries, 1:1, 2:1, and 1:2, conduction velocities increase with tandem increases (the x-axis shows the G_K1_ values, the corresponding G_Na_ value depends on the ratio used) in G_K1_:G_Na_, which is to say that it is G_Na_ that primarily determines conduction velocity.

### Gap junction conductance changes

While the above results establish the main features of changes in excitability due to reciprocal modulation of I_K1_-I_Na_, a third current, I_gj_, is also involved in determining excitability in cardiac tissue. In order to elucidate the effects of changes in cell coupling, the dependence of reciprocal modulation was studied as the gap junction conductance, G_gj_, was varied from 0.1 to 20 μS and the results are summarized in Figure 3. At low values of G_gj_ (0.1 and 0.2 μS), only a limited range of tandem changes in G_K1_–G_Na_ are possible as a result of inexcitability setting in in the fiber for larger values of G_K1_. These limited ranges are difficult to see in the plots of fiber thresholds vs. G_K1_–G_Na_ values in Figure 3A since there is considerable overlap of curves for Ggj = 0.1–0.3 μS; these ranges can, however, be seen in Figure 3B which shows the effects of G_K1_–G_Na_ reciprocal modulation on conduction velocity: the bottom-most curves are for the lowest values of G_gj_.

As G_gj_ is increased, in addition to the threshold being raised, the shapes of the threshold vs. G_K1_–G_Na_ curves change from monotonically increasing (top four curves in Figure 3A) for G_gj_ <1 μS to a biphasic dependence for G_gj_ > 0.5 μS (bottom four curves in Figure 3A).

In general, conduction velocity increases as G_gj_ is increased at any given value of G_K1_–G_Na_ (Figure 3B). On the other hand, the shape of the conduction velocity vs. G_K1_–G_Na_ curves has the opposite trend: for lower G_gj_ values from 0.1 to 0.5 μS, there is a biphasic dependence (lower 3 curves in Figure 3B) on G_K1_–G_Na_ values and this switches to monotonically increasing dependence for higher values of G_gj_.

### Changes in fiber space constant

Unlike short-duration pulses that minimally affect the fast sodium current as used above, longer duration pulses do allow fibers to reach threshold and conduct APs even with large G_K1_ values. Subthreshold long duration pulses allow fibers to approach steady state conditions and were used to estimate the fiber space constant, λ, in a manner similar to that used by Noujaim et al. (2007) and Seidel et al. (2010). As would be expected from long-duration pulses, we see in Figure 4A that the space constant was insensitive to changes in G_Na_ (flat line). Figure 4A also shows that compared to G_Na_, the space constant is very sensitive to changes in G_K1_ and appears to have a $\frac{1}{\sqrt{G_{K1}}}$ dependence as predicted by linear cable theory. Figure 4B shows the results of reciprocal modulation of G_K1_–G_Na_ at different values of G_gj_: these curves are very similar in form to independent modulation of G_K1_ (Figure 4A) indicating that without activation of I_Na_, λ is primarily dependent on G_K1_. Although the results of reciprocal modulation should be identical to independent modulation of G_K1_ alone, for the sake of consistency, these calculations did use reciprocal modulation. Finally, λ increases with increasing G_gj_, and does appear to have the predicted $\sqrt{G_{gj}}$ dependence as well.

## Discussion

This paper examined the functional effects of reciprocal modulation of I_K1_–I_Na_ on electrophysiological characteristics of single cardiac cells and cardiac fibers using an established model of ventricular cells with the goal of determining a teleological reason for the existence of reciprocal modulation. An important finding was the loss of excitability seen with independent modulation of G_K1_ in ventricular fibers (Figure 2E). While the single cell threshold does increase with G_K1_ (Figuer 2D), the effect on the fiber threshold is much more dramatic than would be expected from the cell results. It appears that the combination of electrotonic effects and increases in G_K1_ has a cumulative effect of raising cellular thresholds and decreasing the space constant (Figure 4A) as was also shown by Noujaim et al. (2007, see their supplement). Large I_K1_ channel densities lower the excitability of ventricular tissue to the point where fibers become inexcitable in response to short-duration stimuli. This was not predicted by linear cable theory and these results show an important physiological role for reciprocal modulation in ventricular myocytes. Reciprocal modulation would guarantee a commensurate increase in I_Na_ channels thereby preserving cardiac fiber excitability.

The results of simulations of independent modulation of G_K1_ and reciprocal modulation of G_K1_–G_Na_ appears to suggest a paradoxical effect for I_K1_ upregulation: although I_K1_ by itself would be expected to lower excitability and therefore lower conduction velocity as was shown in Figure 2F, reciprocal modulation results in a parallel upregulation of I_Na_ which is predicted by non-linear cable theory to increase conduction velocity (see Appendix) which was confirmed in this study by independent modulation of G_Na_ in Figure 2C. So which effect dominates? The results of the reciprocal modulation simulations here predict that the G_Na_ effect dominates (Figure 2I): upregulation of G_K1_ is predicted to increase conduction velocity. It turns out that this prediction has already been confirmed: Noujaim et al. (2007), Sekar et al. (2007), and Sekar et al. (2009) found that Kir2.1 upregulation increases conduction velocity. However, their explanation was that Kir2.1 upregulation hyperpolarizes the cell resting membrane potential, thereby increasing sodium channel availability. Figure 2F shows that increasing G_K1_ alone actually decreases conduction velocity and this is in agreement with the experimental results of (Veeraraghavan and Poelzing, 2008) and Veeraraghavan et al. (2016) and with data on reduction of conduction velocity by pinacidil activation of I_K−ATP_. With the discovery of reciprocal modulation (Milstein et al., 2012), we can see that I_K1_ upregulation induces a parallel upregulation of I_Na_ which is the root cause of the increase in conduction velocity. Indeed, Figure 2C shows that independent modulation of G_Na_ has the type of pronounced effect on conduction velocity that is predicted by non-linear cable theory and we see in Figure 2I that with reciprocal modulation of G_K1_ and G_Na_, the G_Na_ effect on conduction velocity dominates. This suggests a reinterpretation of the data in Noujaim et al. (2007), Sekar et al. (2007), Sekar et al. (2009) which were based on 4–6 day cultured cells which have been shown to have greatly reduced I_K1_ (Mitcheson et al., 1996; Christé, 1999). The conduction velocity increases can be understood to be caused by the large and “hidden” upregulation of I_Na_ alone; it is not necessary to resort to the sodium channel availability argument which is unlikely to be valid in cells *in vivo*.

The concept of a fiber space constant is based on linear cable theory and is used to estimate the spatial effect of stimuli and propagating waves. Assuming the ionic current has a linear conductance, we can estimate the length of fiber that will be excited by a local subthreshold stimulus. Cardiac fibers are not continuous cables with linear membrane conductances; they have discontinuous intercellular conductance changes at gap junctions. Furthermore, they have numerous non-linear and time-dependent currents and even the relatively “simple” I_K1_ has a non-linear dependence on voltage (see Methods). Although these caveats preclude a direct application of linear cable theory to compute space constants, the cable theory prediction of the space constant, λ, being proportional to $\sqrt{\frac{G_{gj}}{G_{K1}}}$. appears to hold. A more accurate analysis of the effects on λ, is likely to require subcellular discretization as used by Seidel et al. (2010). The decrease in λ with increasing G_K1_ explains the loss of excitability seen at higher G_K1_ values: an increasing in G_K1_ from the default value of 0.5–1.5 μS halves the value of λ (Figure 4A). The sharp drop in λ with G_K1_ makes it more difficult to bring enough cells to threshold to trigger a propagating AP. While the liminal length has been shown to be proportional to $\sqrt{\frac{G_{K1}}{G_{Na}}}$ for simplified non-near cae models (Jack et al., 1975) the results of this study imply that the liminal length appears to increase much more dramatically with G_K1_ than the predictions of simplified models. The predicted dependence of the liminal length on the ratio of G_K1_ to G_Na_ does explain why reciprocal modulation preserves excitability since tandem changes in these two conductances would leave both their ratio and, therefore, the liminal length intact.

Non-linear cable theory using simplified models (Jack et al., 1975) predicted that conduction velocity, θ, will be proportional the square-root of both G_Na_ and G_gj_: θ ∝ $\sqrt{G_{Na}G_{gj}}$. Similar results were also obtained by Shaw and Rudy (1997, Figures 4, 6) and Veeraraghavan et al. (2016, Figure 7) using different non-linear ionic models and our results are qualitatively consistent with these earlier studies. While Veeraraghavan et al. (2016) also showed that a potassium conductance had a qualitatively similar effect on transverse conduction velocity to that seen here, this conductance had biphasic effect on longitudinal conduction velocity; however, their potassium channel was of the delayed rectifier type and may explain the differences between their results and those in this paper.

Limitations of this study: For one-dimensional or planar AP propagation, the monodomain model used here is generally considered sufficient for the study of cardiac AP propagation while two-dimensional and anisotropic AP propagation may require bidomain models. Mori et al. (2008) showed that the resistance of the intercellular cleft space has a significant effect on AP propagation via ephaptic coupling when gap junction conductance is reduced. These mechanisms were not employed here (also see Dhein et al., 2014) but it is possible, as indicated by the results of Veeraraghavan et al. (2016), that the co-localization of Nav1.5 and Kir2.1 in intercalated disks may have important modulatory effects on currents in the intercellular cleft space which may involve another role for reciprocal modulation. The results of Kucera et al. (2002) and Wei et al. (2016) suggest that computation models that incorporate spatial subcellular localization of ion channels may be useful in explaining phenomena in anisotropic tissue and at the extremes of high G_K1_, low G_Na_, and very low or very high G_gj_. While cardiac cells have spatial variations in channel densities such as pools of Nav channels and calcium fluxes at T-tubules, each cell in cardiac tissue has been shown experimentally (Bu et al., 2009) to be roughly isopotential suggesting that the monodomain cell isopotential model used here is a reasonable one. Such approximations of isopotential intracellular conditions have also been used in ephaptic coupling simulations (Wei et al., 2016).

Cardiac cells assemble ion channel subunits composed of homomeric and heteromeric alpha subunits and heteromeric alpha-beta subunits in endoplasmic reticula and subsequently insert these channels at targeted locations in the cell membrane such as intercalated disks or t-tubules using structural proteins such as SAP97, Ankyrin-G, syntrophin, and dystrophin (Balse et al., 2012). Reciprocal modulation appears to be one way control the densities of I_K1_ and I_Na_ channels. Although the exact mechanism of reciprocal modulation and the exact stoichiometry of I_K1_–I_Na_ channels is unknown at this time, our results show that reciprocal modulation allows better control over excitability in ventricular tissue. Furthermore, it is likely that G_gj_ or gap junction channel density is also probably controlled by analogous cell trafficking and assembly mechanisms to control conduction velocities in ventricular tissue. Cardiac ventricular conduction velocities are usually in the range from 30 to 100 cm/s and the results shown in Figure 3B suggest that G_K1_, G_Na_, and G_gj_ have to stay within a limited and coordinated range of values to accomplish target speeds. Biological data show that these three channel types are co-localized in IDs suggesting the possibility of cooperative behavior between Kir2.1, Nav1.5, and Connexin43 channel proteins.

Lastly, this study yields a second apparently paradoxical result that could be a testable hypothesis. Just as I_K1_ upregulation through reciprocal modulation can be understood to enhance conduction velocity, overexpression of Nav1.5 in cardiac cells will appear to have the counter-intuitive effect of raising the threshold for activation: this is due to the fact that through reciprocal modulation, Nav1.5 upregulation will cause a parallel upregulation of Kir2.1 and, at least in isolated cells (Figure 2G), the I_K1_ effect on threshold is predicted to dominate, thus increasing the cell stimulus threshold. The predicted effect of I_K1_–I_Na_ upregulation on fiber thresholds is more complicated as seen in Figure 2H but nevertheless can be tested *in vitro*. Cardiac cells have evolved a number of mechanisms to keep relative ion channel densities within certain ranges. It is logical that two opposing currents such as I_K1_ and I_Na_ would have to be “balanced” in some way for cells to function properly. Reciprocal modulation may be just one of many such control mechanisms for cardiac excitability.

## Author contribution

AV conceived the experiments, carried them out and wrote the manuscript.

### Conflict of interest statement

The author declares that the research was conducted in the absence of any commercial or financial relationships that could be construed as a potential conflict of interest.
