# Supplementary material for: Reciprocal Modulation of IK1–INa Extends Excitability in Cardiac Ventricular Cells
Source: Front Physiol. 2016 Nov 15;7:542. doi: 10.3389/fphys.2016.00542 (PMC5108932; doi:10.3389/fphys.2016.00542)
Supplement: Supplementary file 2 [file DataSheet2.docx]

# Appendix

## Linear Cable Theory

The ideas of threshold stimulus levels, space constants and liminal lengths were proposed by Rushton (1937) in the context of a linear partial differential equation for electrical activity in excitable (nerve or muscle) fibers:

$C_{m}\frac{\partial V_{m}}{\partial t}=\frac{1}{r_{e}+r_{i}}\frac{\partial^{2}V_{m}}{\partial x^{2}}-I_{ionic}$ (1)

Where C_m_ is the membrane capacitance, V_m_ is the transmembrane potential, r_e_ and r_i_ are, respectively, the resistivities of the external and internal solutions and I_ion_ is the net ionic current through the membrane. Assuming that I_ionic_ is a simple linear ohmic current (I_ionic_ = V_m_/R_m_) where R_m_ is some overall membrane resistance, we would be able to rewrite equation (1) as:

$$R_{m}C_{m}\frac{\partial V_{m}}{\partial t}=\frac{R_{m}}{r_{e}+r_{i}}\frac{\partial^{2}V_{m}}{\partial x^{2}}-I_{ionic}R_{m}$$

which can be rephrased as:

$$\tau_{m}\frac{\partial V_{m}}{\partial t}=\lambda^{2}\frac{\partial^{2}V_{m}}{\partial x^{2}}-V_{m}$$

where τ_m_ is the time constant for charging the membrane capacitance and λ is the fiber space or length constant: $\lambda=\sqrt{\frac{R_{m}}{r_{e}+r_{i}}}$

## Nonlinear Cable Theory

The Hodgkin-Huxley model of ionic currents formulates I_ionic_ in

$C_{m}\frac{\partial V_{m}}{\partial t}=\frac{1}{r_{e}+r_{i}}\frac{\partial^{2}V_{m}}{\partial x^{2}}-I_{ionic}(V_{m},t)$ (2)

as a sum of the individual currents for each ion: $I_{ionic}=\sum_{ion} G_{ion}\left( V_{m},t \right) (V_{m}-E_{ion})$ where G_ion_(V_m_, t) is the voltage- and time-dependent conductance for a particular ion. Thus, the full nonlinear time-dependent cannot be easily simplified to a linear partial differential equation. I_K1_ and I_Na_ are two such ionic currents and a number of other currents are involved as well in the model used in this study.

A number of critical features of excitability depend on I_K1_, I_Na_, and the cardiac intercellular gap junction current, I_gj_, (see Figure 1 and Introduction). One component of excitability that depends on I_K1_ and I_gj_ is the fiber space constant. Assuming that subthreshold stimuli keep a cardiac fiber in the linear cable theory region of operation, we can convert the equation for the space constant to:

$\lambda=\sqrt{\frac{\frac{L_{c}}{G_{K1}}}{r_{e}+\frac{1}{G_{gj}L_{c}}}}$

where *L_c_* is the cell length. By further assuming that the resistivity, *r_e_*, of the extracellular fluid is minimal, we can rewrite the above equation as:

$\lambda=L_{c}\sqrt{\frac{G_{gj}}{G_{K1}}}$.

Other components of excitability include the current stimulus threshold for initiating an action potential (AP), the closely related concept of liminal length and AP conduction velocity all three of which depend on nonlinear fiber properties. The liminal length is the length of fiber at the AP wavefront with sufficient inward depolarizing current such as I_Na_ to overcome the outward repolarizing currents such as I_K1_ in the downstream section of the fiber so that an AP can propagate. The liminal length depends on the nonlinear active properties of I_Na_, the nonlinear properties of I_K1_ and the linear passive properties of intercellular gap junction current, I_gj_. A suprathreshold stimulus is one that depolarizes enough of the fiber beyond the liminal length to allow a propagating AP to occur. Fozzard et al. (1972) showed that the liminal length, LL, in simplified nonlinear models of cardiac fibers is directly proportional to the square root of G_K1_ and inversely proportional to the square root of G_Na_:

LL ∝ $\sqrt{\frac{G_{K1}}{G_{Na}}}$

The linear and nonlinear cable theory predictions are used as a starting point in this study of the effects of I_K1_-I_Na_ modulation.

Simplified models of cardiac fibers have been derived using various simplifying assumptions in the past: Fozzard et al. (1972) applied Rushton’s linear cable theory to estimate stimulus strength-duration curves for Purkinje fibers. Noble (1972) used a steady-state stimulus assumption to derive conditions for threshold phenomena for cases where the ionic current, I_ionic_(V_m_), was not time-dependent and had a simple polynomial dependence on cell membrane potential and showed that successful AP propagation required that the part of the fiber with net inward (negative) current had to overcome the rest of fiber which had net outward (positive) current. This is analogous to the source-sink concept of propagation safety factor in Shaw et al. (1997). Hunter et al. (1975) predicted that the conduction velocity, θ, would be proportional to the square root of the conductance, G_Na_, of I_Na_ and also proportional to the gap junction conductance, G_gj_:

θ ∝ $\sqrt{G_{Na}G_{gj}}$

However, the use of simplified time-independent polynomial voltage dependence to model ionic currents make predictions from simplified models difficult to interpret. Furthermore, cardiac cells have discrete gap junctions connecting cells resulting in discontinuous propagation (Joyner, 1982; Spach et. al, 1987). Joyner (1982) showed that while a uniformly high intracellular conductance value between cell patches does result in the conduction velocity having a square-root dependence on the intracellular conductance as predicted by cable theory and slightly lower-conductance gap junctions also results in similar conduction velocity dependence, once the gap junction conductance is decreased to the point where conduction velocity is less than 10 cm/sec, spatial discontinuities in propagation will result in slower conduction velocities than would be expected from the usual square-root dependence. Spach et al. (1987) showed critical dependence of anisotropic conduction patterns on gap junction conductance.

Simplified models have also been used (Jack *et al.* 1975, Hunter *et al.* 1975, Keener 1991) to predict the dependence of AP thresholds and fiber conduction velocities on ion channel conductances. Nonlinear cable theory was used (Jack *et al.* 1975) to predict that the threshold current, I_th_, would be proportional to the square-root of the fiber intercellular gap junction conductance (G_gj_) and inversely proportional to the square root of the I_K1_ conductance, G_K1_:

I_th_ ∝ $\sqrt{\frac{G_{gj}}{G_{K1}}}$

## Nonlinear Ionic Conductance Models

The nonlinear ionic conductance model used in this study has the general form:

$C_{m}\frac{\partial V_{m}}{\partial t}=G_{gj}\frac{\partial^{2}V_{m}}{\partial x^{2}}-\sum G_{ion}(V_{m},\vec{y})$($V_{m} -E_{ion})$

$\frac{\partial\vec{y}}{\partial t}=\vec{f}\left( V_{m},\vec{y} \right)$ (3)

In the above set of coupled nonlinear differential equations, each ionic conductance ($G_{ion}(V_{m},\vec{y})$) may be a nonlinear function of the local cell membrane potential and ion channel gating variables, $\vec{y}$, which are, in turn, coupled to the cell membrane potential, $V_{m}$ via the nonlinear vector function, $\vec{f}$. These equations are not amenable to analytic solutions and thus prevent *a priori* predictions of responses of cells or tissue to parameter changes, especially when multiple parameters are changed. As a result of the nonlinear differential equations used in cardiac cell models, the exact nature of the dependence of cardiac excitability on ion channel conductances is not easily determined. Many good mathematical models with the same general structure as equation (3) are currently available for cardiac cells. Shaw et al. (1997) used a similar model to the one used in the present study to show that conduction velocity increases with G_Na_ (Fig. 4 in Shaw (1997)) and G_gj_ roughly consistent with the prediction of Hunter et al shown above.

While some general trends predicted by linear and nonlinear cable theory do hold in simulations using such nonlinear ionic conductances, phenomena such as tissue inexcitability were not predicted and thus require the full nonlinear cell models used in this study.
