# Supplementary material for: Reciprocal Modulation of IK1–INa Extends Excitability in Cardiac Ventricular Cells
Source: Front Physiol. 2016 Nov 15;7:542. doi: 10.3389/fphys.2016.00542 (PMC5108932; doi:10.3389/fphys.2016.00542)
Supplement: Supplementary file 3 [file Presentation1.PDF]

Supporting Material for:

Reciprocal modulation of IK1-INa extends excitability in cardiac ventricular cells

Author: Anthony Varghese

Address: Department of Computer Science, University of Wisconsin-River Falls,

River Falls, WI 54022, USA

Correspondence: anthony.varghese@uwrf.edu

Contents:

I. Guinea-Pig Ventricular Cell Model
   a. Schematic view of cell model – Figure S1
   b. Differential Equations
   c. Membrane Ionic Currents
   d. SR calcium handling
   e. List of Parameters and normal values – Table S1
   f. Action potential and time dependent behavior – Figure S2
   g. I_{K1} and I_{Na} responses to suprathreshold stimulus pulse – Figure S3
II. Robustness of Model and Sensitivity to Parameter Values – Figure S4

I. Guinea-Pig Ventricular Cell Model

a. Schematic view of cell model

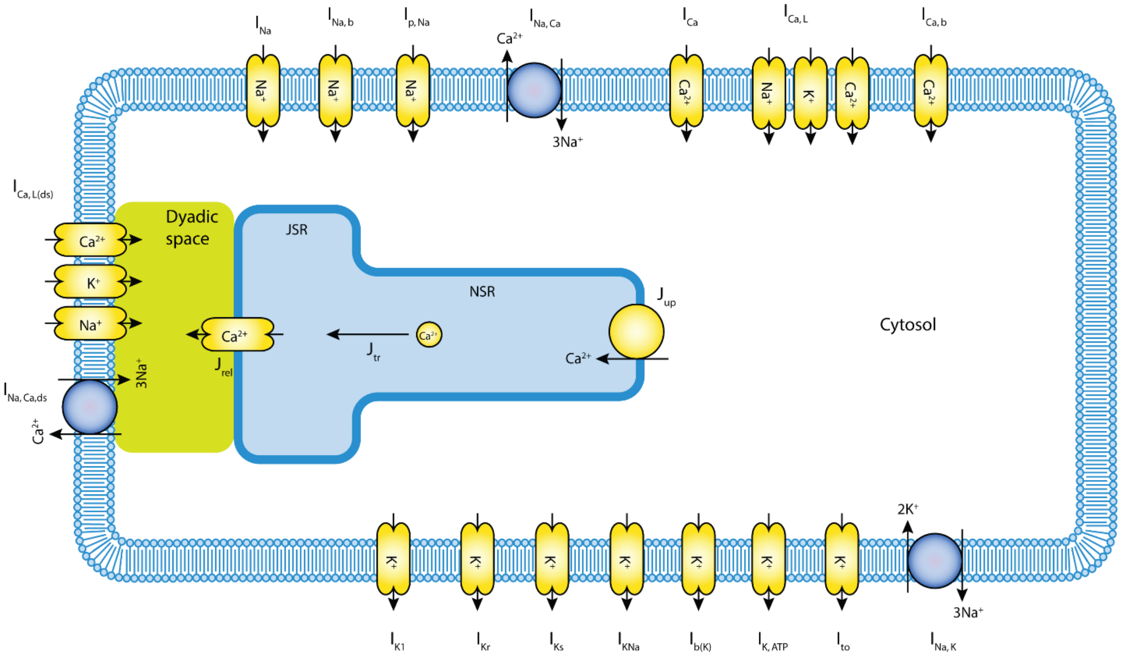

Figure S1: Schematic view of cell model of Noble *et al.* (1998) showing ionic currents due to sodium (Na+), potassium (K+) and calcium (Ca2+) ion channels, Na-K pump and Na-Ca exchanger and intracellular ion fluxes

The equations for each of these currents are listed below.

b. Differential Equations.

The guinea-pig ventricular cell model used in the manuscript comprises 22 differential equations describing the electrical activity and ionic concentration changes in single GP cardiac myocytes (Noble *et al*. 1998).

Equations describing muscle contraction are omitted here as those equations do not influence the electrical activity and thus play no role in the effects of sodium channel block. In addition, we assume that there is no activation of the acetylcholine-sensitive nor ATP-sensitive potassium currents nor that of the stretch-activated currents (Noble *et al*. 1998) and, therefore, these currents are not considered here.

The full equations are provided as a Matlab file accompanying this document. The basic model (not including Na+ channel block) is also available from http://cellml.org and can be simulated using the free COR or OpenCOR software.

1. Membrane Potential, V(t):

$$\dot{V}(t) = \frac{-I_m}{C_m}$$

The total membrane current, I_m, is the sum of the sodium, potassium, and calcium current flowing across the cell membrane into or out of the cytosol:

$$I_m = I_{Na,tot} + I_{K,tot} + I_{Ca,tot} + I_{stim}$$

The total cell membrane sodium current comprises 8 components:

$$I_{Na,tot} = I_{Na} + 3I_{NaK} + I_{b,Na} + I_{p,Na} + I_{CaLNa} + I_{CaLNa,ds} + 3I_{NaCa} + 3I_{NaCa,ds}$$

The total cell membrane potassium current comprises 9 components:

$$I_{K,tot} = I_{K1} + I_{to} + I_{Kr} + I_{Ks} + I_{KNa} + I_{bK} - 2I_{NaK} + I_{CaLK} + I_{CaLK,ds}$$

The total cell membrane calcium current comprises 5 components:

$$I_{Ca,tot} = I_{CaLCa} + I_{CaLCa,ds} + I_{bCa} - 2I_{NaCa} - 2I_{NaCa,ds}$$

There are a total of 19 cell membrane ionic current components in this model. Some currents are common to more than one ion: the sodium-potassium pump current and the sodium-calcium exchanger currents appear in equations for the total sodium, potassium, and calcium membrane currents. The detailed formulations of these ionic currents are described below.

2. Sodium channel activation m(t):

$$\dot{m}(t) = \frac{200(V+41)}{1-e^{-0.1(V+41)}}(1-m) - 8000e^{-0.056(V+66)}m$$

3. Sodium channel inactivation h(t):

- The following equations use the Modulated Receptor model (Hondeghem & Katzung 1977) of block of the sodium channel current. The next six variables describe the fraction of Na channels in:
    - The activated or open state: $A = m^3h$
    - The drug-bound activated state: $A' = m^3h_D$
    - The resting (or deactivated) state: $R = h - A$
    - The drug-bound resting state: $R' = h_D - A'$
    - The inactivated state: $I = 1 - b - h$
    - The drug-bound inactivated state: $I' = b - h_D$
    - [D] is the concentration of the theoretical (lidocaine-like) drug
    - $\alpha_h(V) = 20e^{-0.125(V+75)}$
    - $\beta_h(V) = \frac{2000}{1+320e^{-0.1(V+75)}}$
    - $\alpha_{hD}(V) = 20e^{-0.125(V+75+\Delta V)}$
    - $\beta_{hD}(V) = \frac{2000}{1+320e^{-0.1(V+75+\Delta V)}}$

$$\dot{h}(t) = \alpha_h \mathrm{I} - \beta_h h - (k_r R + k_a \mathrm{A})[D] + (l_r \mathrm{R}' + l_a \mathrm{A}')$$

4. Sodium channel drug bound inactivation $h_D(t)$:

$$\dot{h_D}(t) = \alpha_{hD} \mathrm{I}' - \beta_{hD} h_D + (k_r R + k_a \mathrm{A})[D] - (l_r \mathrm{R}' + l_a \mathrm{A}')$$

5. Sodium channel fraction of drug bound channels b(t):

$$\dot{b}(t) = (k_r R + k_a \mathrm{A} + k_i \mathrm{I})[D] - (l_r \mathrm{R}' + l_a \mathrm{A}' + l_i \mathrm{I}')$$

6. L-type Calcium channel activation d(t):

$$\dot{d}(t) = \frac{90(V+19)}{1 - e^{-(V+19)/4}}(1-d) - \frac{36(V+19)}{e^{(V+19)/10} - 1} d$$

7. L-type Calcium channel inactivation f(t):

$$\dot{f}(t) = \frac{0.6}{1 + e^{-(V+34)/4}} \left[\frac{120[Ca]_i + k_{cachoff}}{[Ca]_i + k_{cachoff}}\right](1-f) - \frac{6.25(V+34)}{e^{(V+34)/4} - 1} f$$

8. Rapid delayed rectifier potassium channel $x_{r1}(t)$:

$$\dot{x_{r1}}(t) = \frac{50}{1 + e^{-(V-5)/9}}(1 - x_{r1}) - 0.05 e^{-(V-20)/15} x_{r1}$$

9. Rapid delayed rectifier potassium channel $x_{r2}(t)$:

$$\dot{x_{r2}}(t) = \frac{50}{1 + e^{-(V-5)/9}}(1 - x_{r2}) - 0.4 e^{-\left(\frac{V+30}{30}\right)^3} x_{r2}$$

10. Slow delayed rectifier potassium channel activation $x_s(t)$:

$$\dot{x_s}(t) = \frac{14}{1 + e^{-(V-40)/9}}(1 - x_s) - e^{-V/45} x_s$$

11. Transient outward potassium current activation r(t):

$$\dot{r}(t) = 333\left(\frac{1}{1 + e^{-\left(\frac{V+4}{5}\right)}} - r\right)$$

12. Transient outward potassium current inactivation s(t):

$$\dot{s}(t) = 0.033 e^{-V/17}(1-s) - \frac{33}{1 + e^{-(V+10)/8}} s$$

13. Internal sodium concentration $[Na^+]_i(t)$: $\dot{[Na_i]}(t) = -I_{Na,tot}/V_i F$
14. Internal potassium concentration $[K^+]_i(t)$: $\dot{[K_i]}(t) = -I_{K,tot}/V_i F$

15. Internal calcium concentration $[Ca^{2+}]_i(t)$:

$$[\dot{Ca_i}](t) = -I_{Ca,tot}/V_iF - I_{up} + I_{rel}\,V_{rel}/V_i - [\dot{Ca}]_{calmod} - [\dot{Ca}]_{troponin} + D_{Ca}([Ca]_{ds} - [Ca]_i)V_{ds}$$

16. Dyadic space calcium concentration $[Ca^{2+}]_{ds}(t)$:

$$[\dot{Ca}_{ds}](t) = \frac{-(I_{CaLCads} - 2I_{NaCads})}{2V_{ds}F} - D_{Ca}([Ca]_{ds} - [Ca]_i)$$

17. SR Calcium uptake pool $[Ca^{2+}]_{up}(t)$: $[\dot{Ca}]_{up} = \frac{V_i}{V_{SRup}} I_{up} - I_{tr}$

18. SR Calcium release pool $[Ca^{2+}]_{rel}(t)$: $[\dot{Ca}]_{rel} = \frac{V_{up}}{V_{rel}} I_{tr} - I_{rel}$

19. Cytosolic calmodulin-bound calcium $[Ca2+]_{calmod}(t)$:

$$[\dot{Ca}]_{calmod} = 10^5(C_{calmodulin} - [Ca]_{calmod})[Ca]_i - 50[Ca]_{calmod}$$

20. Cytosolic troponin-bound calcium $[Ca2+]_{troponin}(t)$:

$$[\dot{Ca}]_{troponin} = \alpha_{trop}\left(C_{trop} - [Ca]_{troponin}\right)[Ca]_i - \beta_{trop}[Ca]_{troponin}$$

21. Activator fraction $f_{activator}(t)$:

$$\dot{f}_{activator}(t) = \left(\left(1 - f_{activator} - f_{product}\right)(500{x_{SRRel}}^2) - f_{activator}(500{x_{SRRel}}^2 + 60)\right)\,\rho$$

$$\rho = 1 + \frac{4}{1 + e^{V+50}}$$

$$x_{SRRel} = \frac{[Ca]_i}{[Ca]_i + k_{mCacyt}} + \left(1 - \frac{[Ca]_i}{[Ca]_i + k_{mCacyt}}\right)\frac{[Ca]_{ds}}{[Ca]_{ds} + k_{mCads}}$$

22. Product fraction $f_{product}(t)$:

$$\dot{f}_{product}(t) = \left(f_{activator}(500{x_{SRRel}}^2 + 60) - f_{product}\right)\rho$$

c. Membrane Ionic Currents:

1. Fast inward sodium current, I_{Na}: $I_{Na} = G_{Na}(V - E_{mh})m^3h$

$$E_{mh} = \frac{RT}{F}\,ln\left(\frac{[Na]_o + 0.12[K]_o}{[Na]_i + 0.12[K]_i}\right)$$

2. Persistent sodium current, I_{pNa}: $I_{pNa} = G_{pNa}\frac{(V-E_{Na})}{1+e^{-\left(\frac{V+52}{8}\right)}}$
3. Inward rectifier I_{K1}: $I_{K1} = G_{K1}\frac{[K]_o}{[K]_o+k_{mk1}}\frac{(V-E_K)}{1+e^{\frac{(V-E_K-10)}{RT/2F}}}$

$$E_K = \frac{RT}{F}ln\left(\frac{[K]_o}{[K]_i}\right)$$

4. Rapid component of delayed rectifier, I_{kr}: $I_{Kr} = (G_{Kr1}x_{r1} + G_{Kr2}x_{r2})\frac{(V-E_K)}{1+e^{\frac{(V+9)}{22.4}}}$
5. Slow component of delayed rectifier, I_{ks}: $I_{Ks} = G_{Ks}x_s^2(V - E_{Ks})$

$$E_{Ks} = \frac{RT}{F}ln\left(\frac{[K]_o + P_{KNa}[Na]_o}{[K]_i + P_{KNa}[Na]_i}\right)$$

6. Background potassium current, I_{bK}: $I_{bK} = G_{bK}(V - E_K)$
7. Transient outward potassium current: $I_{to} = G_{to}\ r\ s\ (V - E_K)$
8. Sodium-sensitive potassium current, I_{KNa}: $I_{KNa} = G_{KNa}\frac{[Na]_i}{k_{mKNa}+[Na]_i}(V - E_K)$
9. Sodium-potassium pump current, I_{NaK}:

$$I_{NaK} = I_{NaKmax}\frac{[K]_o}{k_{mK} + [K]_o}\frac{[Na]_i}{k_{mNa} + [Na]_i}$$

10. Background sodium current, I_{bNa}: $I_{bNa} = G_{bNa}(V - E_{Na})$

$$E_{Na} = \frac{RT}{F}ln\left(\frac{[Na]_o}{[Na]_i}\right)$$

L-type Calcium current (I_{CaL}):

Goldmann-Hodgkin-Katz terms for ionic permeation:

Calcium component G_{LCa}: $G_{LCa} = 2P_{Ca}\frac{\frac{V-50}{RT/2F}}{1-e^{\frac{-(V-50)}{RT/2F}}}\left[[Ca]_i e^{\frac{50}{RT/2F}} - [Ca]_o e^{\frac{-(V-50)}{RT/2F}}\right]$

Potassium component G_{LK}: $G_{LK} = P_{Ca}P_{CaK}\frac{\frac{V-50}{RT/F}}{1-e^{\frac{-(V-50)}{RT/F}}}\left[[K]_i e^{\frac{50}{RT/F}} - [K]_o e^{\frac{-(V-50)}{RT/F}}\right]$

Sodium component G_{LNa}: $G_{LNa} = P_{Ca}P_{CaNa}\frac{\frac{V-50}{RT/F}}{1-e^{\frac{-(V-50)}{RT/F}}}\left[[Na]_i e^{\frac{50}{RT/F}} - [Na]_o e^{\frac{-(V-50)}{RT/F}}\right]$

I_{CaL} current into cytosolic space:

Gating of channels in non-dyadic space, g_{cyt}: $g_{cyt} = d(1 - f)\frac{k_{cachoff}}{k_{cachoff}+[Ca]_i}$

11. Cytosolic Calcium component, I_{caLCa}: $I_{CaLCa} = g_{cyt} G_{LCa}(1 - f_{CaL})$
12. Cytosolic Potassium component, I_{CaLK}: $I_{CaLK} = g_{cyt} G_{LK}(1 - f_{CaL})$
13. Cytosolic Sodium component, I_{CaLNa}: $I_{CaLNa} = g_{cyt} G_{LNa}(1 - f_{CaL})$

L-type Calcium current into the Dyadic space:

Gating of channels in the dyadic space, g_{ds}: $g_{ds} = d(1 - \mathrm{f}) \frac{k_{dsoff}}{k_{dsoff} + [Ca]_{ds}}$

14. Dyadic space Calcium component, $I_{CaLCads}$: $I_{CaLCads} = g_{ds} G_{LCa} f_{CaL}$
15. Dyadic space Potassium component, I_{CaLKds}: $I_{CaLKds} = g_{ds} G_{LK} f_{CaL}$
16. Dyadic space Sodium component, $I_{CaLNads}$: $I_{CaLNads} = g_{ds} G_{LNa} f_{CaL}$
17. Background calcium current, I_{bCa}:$I_{bCa} = G_{bCa}(V - E_{Ca})$

$$E_{Ca} = \frac{RT}{2F} ln\left(\frac{[Ca]_o}{[Ca]_i}\right)$$

18. Dyadic-space Sodium-Calcium Exchanger current, $I_{NaCa,ds}$: $I_{CaLNa,ds} = k_{NaCa} \frac{N_{ds}}{D_{ds}} f_{NaCaX}$

$$N_{ds} = e^{\frac{\gamma(n_{NaCa}-2)V}{RT/F}} [Na]_i^{\,n_{NaCa}} [Ca]_o - e^{\frac{(\gamma-1)(n_{NaCa}-2)V}{RT/F}} [Na]_o^{\,n_{NaCa}} [Ca]_{ds}$$

$$D_{ds} = \left(1 + \ d_{NaCa}\left([Na]_i^{\,n_{NaCa}} [Ca]_o + [Na]_o^{\,n_{NaCa}} [Ca]_{ds}\right)\right)\left(1 + \frac{[Ca]_{ds}}{0.0069}\right)$$

19. Non-dyadic space Sodium-Calcium Exchanger current, I_{NaCa}:

$$I_{NaCa} = k_{NaCa} \frac{N_{cyt}}{D_{cyt}} (1 - f_{NaCaX})$$

$$N_{cyt} = e^{\frac{\gamma(n_{NaCa}-2)V}{RT/F}} [Na]_i^{\,n_{NaCa}} [Ca]_o - e^{\frac{(\gamma-1)(n_{NaCa}-2)V}{RT/F}} [Na]_o^{\,n_{NaCa}} [Ca]_i$$

$$D_{cyt} = \left(1 + \ d_{NaCa}\left([Na]_i^{\,n_{NaCa}} [Ca]_o + [Na]_o^{\,n_{NaCa}} [Ca]_i\right)\right)\left(1 + \frac{[Ca]_i}{0.0069}\right)$$

d. Sarcoplasmic Reticulum (SR) membrane currents and compartment fluxes

1. SR Calcium pump or uptake current, I_{up}:

$$I_{up} = \frac{\alpha_{up}[Ca]_i - \beta_{up}[Ca]_{up} \frac{k_{cyca} k_{xcs}}{k_{srca}}}{[Ca]_i + \ [Ca]_{up} \frac{k_{cyca} k_{xcs}}{k_{srca}} + k_{cyca} k_{xcs} + k_{cyca}}$$

2. SR calcium uptake to release translocation, I_{tr}: $I_{tr} = 50([Ca]_{up} - [Ca]_{rel})$
3. SR calcium release, I_{rel}: $I_{rel} = \left(\left(\frac{f_{activator}}{f_{activator}+0.25}\right)^2 K_{mCa2} + K_{leak}\right)[Ca]_{rel}$

e. Model Parameters

C_m	95 pF	Membrane capacitance – used in differential equation 1
T	310 Kelvin	Temperature
G_{Na}	0.5 μSiemens	Fast sodium conductance
[D]	0-500 μM	Na channel blocker concentration
k_r	0.4 $(mM\ s)^{-1}$	Binding rate for drug to Na channel resting state
k_a	47500 $(mM\ s)^{-1}$	Binding rate for drug to Na channel activated state
k_i	4750 $(mM\ s)^{-1}$	Binding rate for drug to Na channel inactivated state
l_r	7 s^{-1}	Unbinding rate for drug from Na channel resting state
l_a	200 s^{-1}	Unbinding rate for drug from Na channel activated state
l_i	5 s^{-1}	Unbinding rate for drug from Na channel inactivated state
ΔV	35 mV	Voltage shift in Na channel drug-bound inactivation
$[Na^+]_o$	140 mM	Extracellular sodium
$[K^+]_o$	5.4 mM	Extracellular potassium
$[Ca^{2+}]_o$	2 mM	Extracellular calcium
G_{K1}	0.5 μS	Inward rectifier conductance

k_{mk1}	10 mM	Inward rectifier potassium Km
G_{Kr1}	0.0028 μS	Rapid delayed rectifier 1 conductance
G_{Kr2}	0.0017 μS	Rapid delayed rectifier 2 conductance
G_{Ks}	0.0026 μS	Slow delayed rectifier conductance
P_{KNa}	0.03 (no units)	Na fraction of slow delayed rectifier
G_{bK}	0.0003 μS	Background K current conductance
G_{to}	0.005 μS	Transient outward K conductance
G_{KNa}	0.001 μS	Na-activated K current conductance
k_{KNa}	20 mM	Km for Na-activation of I_{KNa}
I_{NaKmax}	0.7 nA	Max Na-K pump current
k_{mk}	1 mM	Km for Ko in Na-K pump
k_{mNa}	40 mM	Km for Nai in Na-K pump
G_{bna}	0.0006 μS	Background Na current conductance
G_{pna}	0.0051 μS	Persistent sodium current conductance
P_{Ca}	1.0 nA/mM	Permeability of L-type Ca channel
P_{CaK}	0.001	Potassium comp of L-type ch current
P_{CaNa}	0.010	Sodium comp of L-type ch current

f_{CaL}	1	Fraction of L-type ch in dyadic space
G_{bCa}	0.00025	Background calcium current conductance
f_{NaCaX}	0.001	Fraction of NaCa exchangers in dyadic space
k_{naca}	$5(10)^{-4}$ nA	Na Ca exchanger rate
γ	0.2	Na Ca exchanger Voltage dependence
d_{naca}	0	Na Ca exchanger denominator factor
α_{up}	0.4 mM/s	Activation of SR release
β_{up}	0.03 mM/s	Inactivation of SR release
k_{cyca}	0.0003 mM	SR pump activation
k_{srca}	0.5 mM	SR pump activation
k_{xcs}	0.4 mM	SR pump activation
k_{mrel}	250 1/s	Rate of activated SR release
K_{leak}	0.05 1/s	Rate of leak SR release
R_{decay}	20 1/s	Rate of inactiv dyadic space L-type Ca ch
k_{decay}	100 ms	Rate of diffusion between dyadic space and cytosolic Ca
radius	12 μm	Average radius of myocyte cross-section
length	74 μm	Myocyte length

V_{ecs}	0.4	Fraction of cell volume taken by extracellular space
V_{up}	0.01	Fractional Volume of SR uptake pool
V_{rel}	0.1	Fractional Volume of SR release pool
V_{ds}	0.1	Fraction of Vcell occupied by dyadic space
D_{Ca}	0.01	Diffusion between dyadic space and cytosol
C_{calmod}	0.02 mM	Max Ca bound to calmodulin
C_{trop}	0.05 mM	Max Ca bound to troponin
α_{calmod}	1e5 $(mM\ s)^{-1}$	Activation of Ca bound to calmodulin
α_{trop}	1e5 $(mM\ s)^{-1}$	Activation of Ca bound to troponin
β_{calmod}	50 s^{-1}	Inactivation of Ca bound to calmodulin
β_{trop}	200 s^{-1}	Inactivation of Ca bound to troponin
$k_{cachoff}$	0.01 mM	Km for cytosolic Ca in Ca inactivation of L-type Ca channels
k_{dsoff}	0.001 mM	Km for dyadic space Ca in Ca inactivation of L-type Ca channels
k_{mcacyt}	0.0005 mM	Km for cytosolic Ca in Ca release
k_{mcads}	0.01 mM	Km for dyadic space Ca in Ca release
G_{gj}	10 μS	Longitudinal gap junction conductance between adjacent cells

Constants:

F	96485.3415 Coul/mol	Faraday constant
R	8314.472 mJoule/(mol K)	Universal gas constant
n_{NaCa}	3	Na Ca exchanger stoichiometry

Derived Parameters:

V_{cell}	π radius^2 length	Total cell volume
V_i	V_{cell} (1 - V_{ecs} - V_{up} - V_{rel} -V_{ds})	Intracellular volume
V_{SRup}	V_{cell} V_{up}	SR uptake pool volume

f. Reconstruction of the Guinea-Pig ventricular cell action potential

The above equations and parameters result in an action potential, gating variable changes, ionic currents, calcium concentrations as shown in Figure S2.

Under control conditions a suprathreshold stimulus activates the sodium current causing "phase 0" depolarization (Figure S2A) of the cell membrane; this, in turn, activates and inactivates the voltage-gated L-type calcium current (Figure S2 B and C) during the plateau phase of the action potential; the influx of calcium triggers further release of calcium from the sarcoplasmic reticulum (Figure S2 F) causing cytosolic calcium to rise (Figure S2E). The sustained depolarization during plateau phase activates voltage-gated potassium channels (Figure S2D) causing cell membrane repolarization. Changes in cytosolic calcium trigger inactivation of I_{CaL} well beyond phase 3 repolarization.

Figure S2: *In silico* time-behavior of cell components under control conditions ([Drug] = 0)

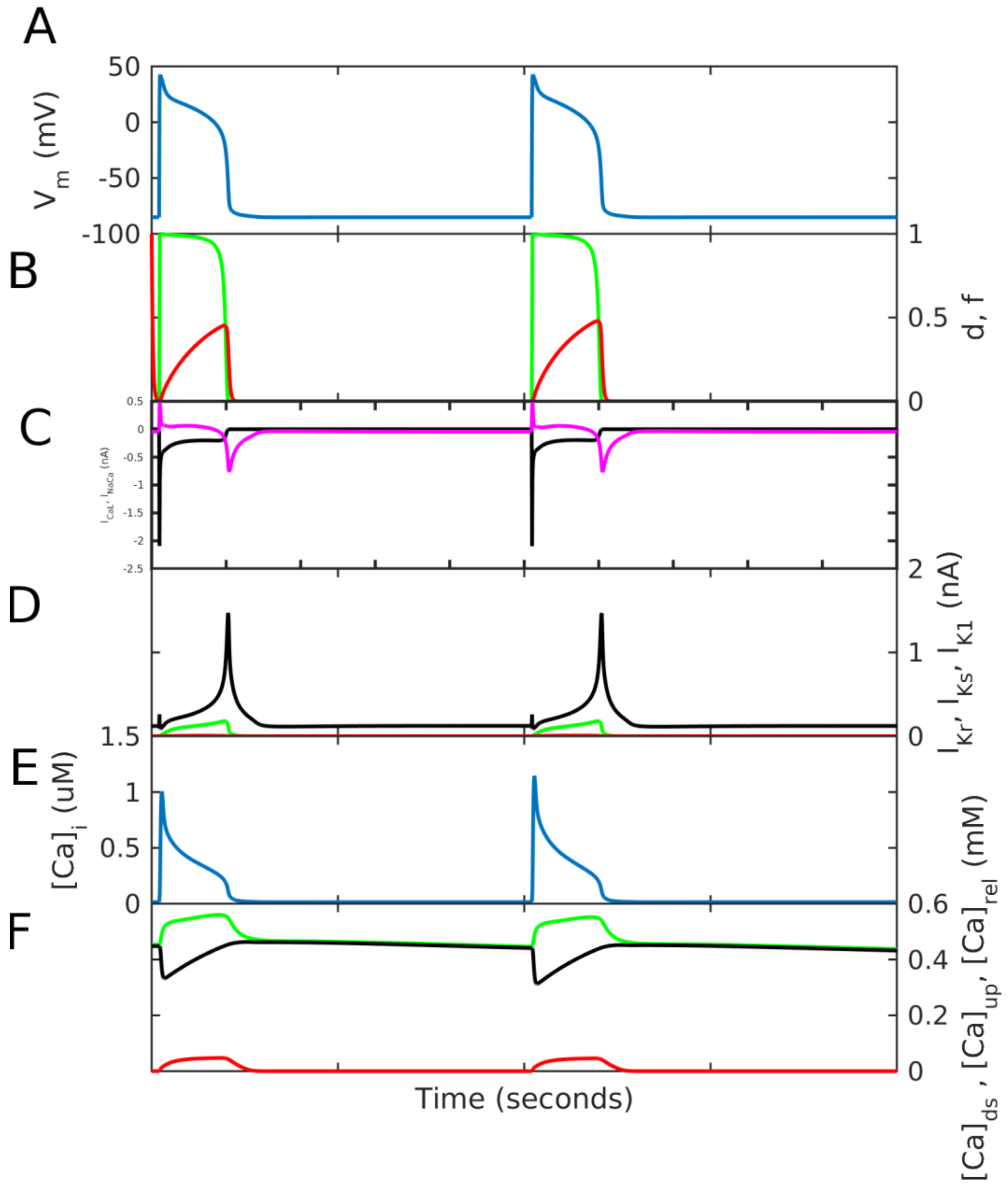

A: Cell membrane potential (V_m) changes due to a super-threshold stimulus administered 100 milliseconds after the start of the simulation.

B: Voltage-dependent activation (green) and inactivation (red) gates of the L-type Ca current, I_{CaL}.

C: I_{CaL} (black) and the Na-Ca exchanger (I_{NaCa} in magenta) currents.

D: Three outward potassium currents: the delayed rectifier I_{Kr} (green) and I_{Ks} (red) currents and the inward rectifier I_{K1} current (black).

E: Cytosolic calcium concentration changes during an action potential.

F: Changes in dyadic space (red) and SR uptake (green) and release pool (black) calcium concentrations.

g. I_{K1} and I_{Na} responses in a suprathreshold stimulus

The sequence of events in response to a 200 μsec suprathreshold stimulus pulse administered starting at the 20 msec time point can be seen in Figure S3 in an expanded time scale. The stimulus depolarizes the cell to about -50 mV and the upstroke of the action potential occurs approximately 1.5 milliseconds later. The sodium current (I_{Na}) does not inactivate (red curve in Figure S3 C) until the upstroke of the action potential is well established. During the 1 millisecond before the upstroke of the action potential, the magnitudes of I_{Na} and I_{K1} are comparable – about 1-1.5 nA – and under control conditions (with no drug) the upstroke of the action potential is accompanied by a larger sodium influx (Figure S3D) and a decline in I_{K1} due to the rectification properties of this current.

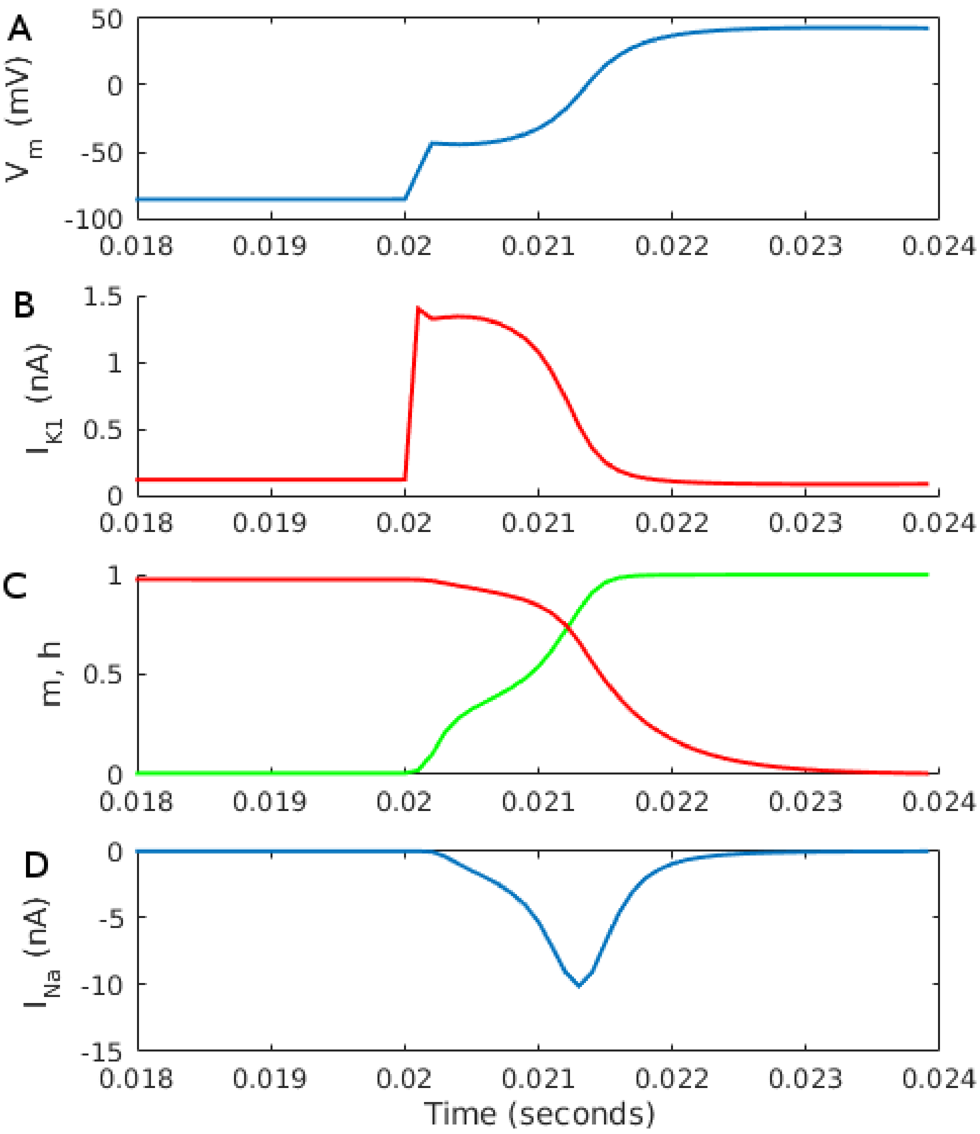

Figure S3: Expanded time-scale of ionic currents during a stimulus and the phase 0 depolarization under control conditions ([Drug] = 0).

A: Changes in the membrane potential, V_m.

B: The inward rectifier current (I_{K1}) is activated by the changes in V_m during the stimulus and declines once the AP upstroke occurs.

C: The activation "m" gate (green) and the inactivation "h" gate (red) during the AP upstroke. The h gate stays close to 1 while the m gate starts opening.

D: The fast inward sodium current (I_{Na}) during the upstroke of the action potential.

II. Robustness of Model and Sensitivity to Parameter Values

The analysis of sensitivity of solutions of differential equations to parameters reveals much about the robustness of observed phenomena. In addition to examining changes in behavior of solutions, sensitivity analysis can also be used to show stability of solutions.

a. Local sensitivity analysis

Local sensitivity was computed using two methods. The "internal numerical differentiation" method of Bock (1981) [3] is the faster technique while the "iterative approximation based on directional derivatives" method of Maly and Petzold (1996) [10] is more accurate. The system of 22 differential equations listed above were integrated for a single action potential with the drug concentration set to 0. The 22 states were augmented with a system of 1408 sensitivity factors (the sensitivities of the 22 state variables to each of the 64 parameters listed above) to yield a total of 1430 values at each point in time during the action potential.

From this data, the 3 largest (absolute value) sensitivity terms were found and listed below for each local sensitivity run:

	3 parameters with highest sensitivity	
State	Internal Numerical Differentiation	Iterative Directional Derivative
d	C_m, k_{cyca}, g_{bCa}	C_m, k_{cyca}, g_{bCa}
f	C_m, k_{cyca}, g_{bCa}	C_m, k_{cyca}, g_{bCa}
$f_{activator}$	C_m, k_{cyca}, g_{bK}	C_m, k_{mCacyt}, g_{bCa}
$f_{product}$	C_m, k_{cyca}, g_{bK}	C_m, k_{cyca}, k_{mCacyt}
h	C_m, k_{cyca}, g_{bCa}	C_m, k_{cyca}, g_{bCa}
m	C_m, k_{cyca}, g_{bCa}	C_m, k_{cyca}, g_{bCa}
$[Ca]_{Calmod}$	C_m, k_{cyca}, g_{bCa}	C_m, k_{cyca}, g_{bCa}
$[Ca]_{troponin}$	C_m, k_{cyca}, g_{bCa}	C_m, k_{cyca}, g_{bCa}

$[Ca]_{ds}$	C_m, k_{cyca}, k_{mf2ds}	C_m, k_{cyca}, k_{mf2ds}
$[Ca]_i$	C_m, k_{cyca}, g_{bCa}	C_m, k_{cyca}, g_{bCa}
$[Ca]_{rel}$	C_m, k_{cyca}, g_{bK}	C_m, k_{cyca}, g_{bCa}
$[Ca]_{up}$	C_m, k_{cyca}, g_{bCa}	C_m, k_{cyca}, k_{mf2ds}
$[K]_i$	C_m, k_{cyca}, g_{bCa}	g_{bCa}, C_m, k_{cyca}
$[Na]_i$	C_m, k_{cyca}, g_{bCa}	C_m, k_{cyca}, g_{bCa}
V_m	C_m, k_{cyca}, g_{bCa}	C_m, k_{cyca}, g_{bCa}
x_{r1}	C_m, k_{cyca}, g_{bCa}	C_m, k_{cyca}, g_{bCa}
x_{r2}	C_m, k_{cyca}, g_{bCa}	C_m, k_{cyca}, g_{bCa}
x_s	C_m, k_{cyca}, g_{bCa}	k_{cyca}, C_m, k_{mCacyt}
r	k_{cyca}, C_m, g_{bCa}	C_m, k_{cyca}, g_{bCa}
s	C_m, k_{cyca}, g_{bCa}	C_m, k_{cyca}, g_{bCa}
h_D	[D], k_{mCads}, k_{mCacyt}	[D], k_{mCads}, k_{mCacyt}
B	[D], k_{mCads}, k_{mCacyt}	[D], k_{mCads}, k_{mCacyt}

The two methods produced slightly different quantitative data but the lists of most sensitive parameters were identical. Note that the nominal value of the drug concentration [D] was 0 and the above results show that small variations in this parameter can have very significant changes in a number of state variables. It is important to remark that the voltage-dependent kinetic terms were considered constant and not varied; it is likely that if these were to be considered as parameters, they would certainly be the most sensitive ones for the gating variables. All computations were performed in Matlab using the ode15s stiff system solver through the sens_ind and sens_sys packages (http://www.mathworks.com/matlabcentral/fileexchange/1480-sensitivity-analysis-for-odes-and-daes)

Figure S4: Examples of local sensitivity analysis results in a simulation under control conditions (no drug) and BCL of 500 msec.

A: Changes in cell membrane potential (V_m).

B: Cytosolic calcium concentration changes

C: Sensitivity of Vm with respect to cell membrane capacitance, C_m, (blue curve) and with respect to the maximum sodium conductance, G_{Na} (magenta).

D: Sensitivity of $[Ca]_i$ with respect to C_m (blue) and k_{mCacyt} (magenta).

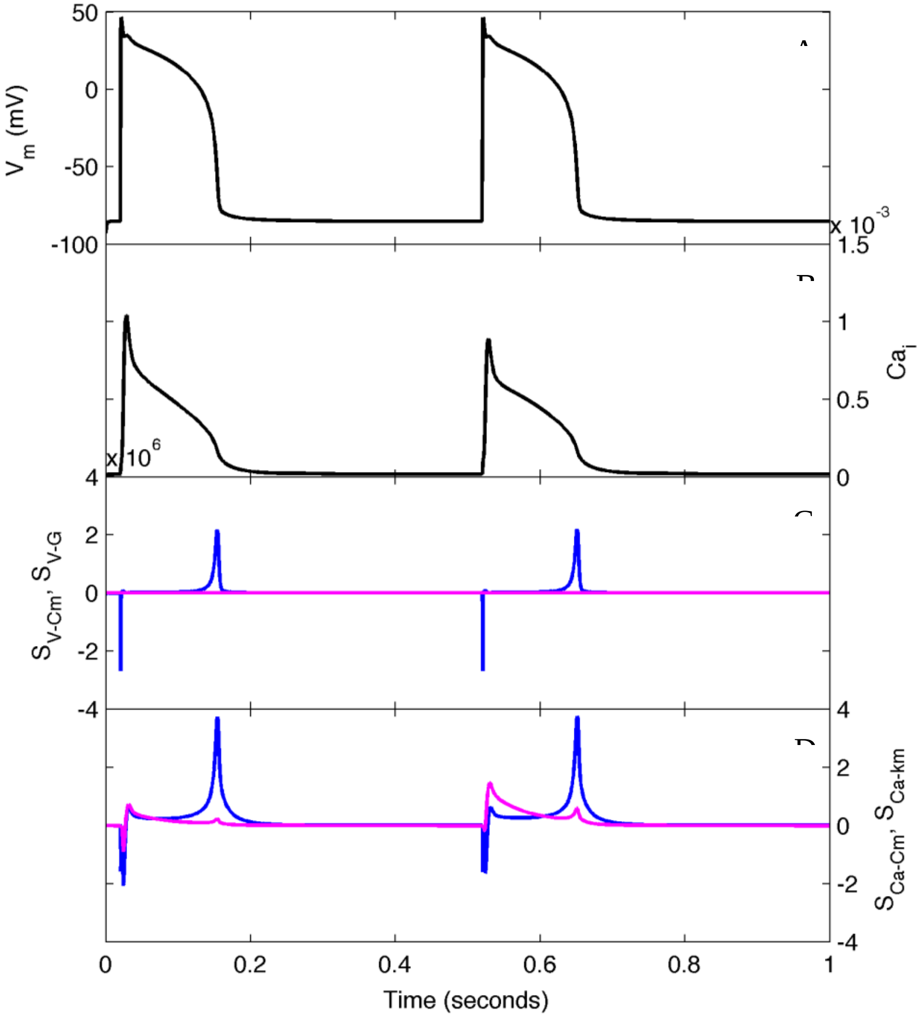

A sampling of the results of local sensitivity computations is shown in Figure S4 and reveal much about the parameter dependence of these models. The full data set obtained from the sensitivity computations – 1430 sensitivities – is too large to present completely here and we plot 4 examples in Figure S4. Changes in V_m and $[Ca]_i$ are shown in Figure S4A-B for reference and the time-evolution of corresponding sensitivities are shown in S4 C-D.

The sensitivity of V_m to the cell membrane capacitance, C_m, is remarkable in that it is rather large. This can be attributed to the fact that C_m appears in the denominator of the differential equation for V_m (differential equation 1) and determines the time constant for charging the cell membrane. It is also remarkable that this sensitivity occurs primary when Vm changes – during the upstroke of the AP and during phase 3 repolarization. On the other hand, V_m is almost insensitive to the maximal conductance, G_{Na}, of the fast sodium current even during the upstroke of the action potential and the repolarization phase.

The sensitivity of $[Ca]_i$ to C_m is many orders of magnitude smaller which can be attributed to the fact that $[Ca]_i$ is not directly related to C_m – it is related to C_m through changes in V_m.

Yet, $[Ca]_i$ is more sensitive to C_m than to k_{mCacyt} which does appear in the differential equation for $[Ca]_i$.

1 References

[1] HG Bock, "Numerical Treatment of inverse problems in chemical reaction kinetics," in *Modelling of Chemical Reaction Systems*. Heidelberg: Springer, 1981.

[2] L M Hondeghem and B G Katzung, "Time- and voltage-dependent interactions of antiarrhythmic drugs with cardiac sodium channels.," *Biochim. Biophys. Acta.*, vol. 472, pp. 373-398, 1977.

[3] T Maly and L Petzold, "Numerical methods and software for sensitivity analysis of differential-algebraic systems," *Appl. Num. Math*, vol. 20, pp. 57-79, 1996.

[4] D Noble, A Varghese, P Kohl, and P Noble, "Improved guinea-pig ventricular cell model incorporating a diadic space, IKr and IKs, and length- and tension-dependent processes.," *Can. J. Cardiol.*, vol. 14, no. 1, pp. 123-134, 1998.
